# The Relationship Between Edema and Body Functions in Patients With Chronic Kidney Disease: A Preliminary Study

**DOI:** 10.7759/cureus.27118

**Published:** 2022-07-21

**Authors:** Ryota Takase, Takeshi Nakata, Kohei Aoki, Mitsuhiro Okamoto, Akihiro Fukuda, Naoya Fukunaga, Koro Goto, Takayuki Masaki, Hirotaka Shibata

**Affiliations:** 1 Physical Medicine and Rehabilitation, Oita University, Oita, JPN; 2 Endocrinology Metabolism, Rheumatology, and Nephrology, Oita University, Oita, JPN

**Keywords:** body composition, chronic kidney disease (ckd), ecw/tbw, physical function, edema

## Abstract

Introduction: Chronic kidney disease (CKD) is known to be a risk factor for falls. In addition, numerous factors such as impaired body balance and loss of muscle mass were reported as risk factors for falls. Patients with CKD often have edema in their lower extremes. In Japan, edema, as well as physical factors, are listed as fall assessment items. Little is known about the relation between body functions and edema in patients with CKD. Thus, we conducted a multivariate regression analysis to investigate the factors related to knee extension muscle strength and dynamic balance in motion (TUG).

Materials and methods: Thirty patients with CKD participated in this study. The basic characteristics were sex, age, blood pressure, body mass index (BMI), and medications. The laboratory data were estimated glomerular filtration rate (eGFR), hemoglobin (Hb), and C-reactive protein (CRP). Edema and muscle mass was measured by using InBody S10 (Inbody Japan Inc., Tokyo, Japan). The balance function while standing at rest and motion was measured as the total trajectory length of the center of gravity and the index of postural stability (IPS) using a kinetogravicorder 7100 (Anima Inc., Tokyo, Japan). Dynamic balance was assessed by the timed up & go (TUG) test. Knee extension muscle strength was measured by the Micro Total Analysis System (μ-Tas) F-1 (Anima Inc., Tokyo, Japan) test. Nutritional assessment was measured by the geriatric nutritional risk index (GNRI). Activities of daily living were measured using the functional independence measure (FIM). We conducted a multivariate regression analysis to investigate the factors related to knee extension muscle strength and dynamic balance in motion.

Results: Extracellular water/total body water (ECW/TBW) was not significantly correlated with balance at rest and IPS. The ECW/TBW was associated with knee extension muscle strength, TUG, albumin (Alb), Hb, and GNRI with statistical significance. After adjusting for sex and age, knee extension muscle strength was associated with ECW/TBW and TUG (p=0.044). The TUG was also associated with ECW/TBW after being adjusted for age and sex (p=0.046).

Conclusion: Patients with CKD who have edema may have decreased knee extensor strength and body balance function. Investigation of knee extension muscle strength and the body balance test in addition to the presence of leg edema at the time of physical examination may help predict a functional decline in CKD patients.

## Introduction

In Japan, the number of patients with chronic kidney disease (CKD) is estimated to be 13,560,000 [1], and its incidence is continually increasing [2]. It has been reported that CKD patients tend to have a relative decrease in physical function [3]. Moreover, the physical function of CKD patients decreases as CKD progresses, which may cause increased mortality [4,5].

Brandon et al. reported that CKD patients aged 65 years and older had a higher risk of falling compared to healthy individuals in the same age group without CKD (odds ratio (OR) 1.50, 95% confidence interval (CI) 1.27-1.78) [6]. Additionally, hospitalization due to falls is correlated with mortalities for hemodialysis patients. A systematic review examining the causes of falls among elderly individuals reported that dynamic balance and walking disorders are risk factors for falls [7]. In addition, numerous factors such as impaired body balance and loss of muscle mass were reported as risk factors for falls [8]. In Japan, edema, as well as physical factors such as muscle weakness and poor balance, are listed as fall assessment items [9,10]. However, the mechanism by which edema is related to physical functions remains unknown.

Although edema is easily detected by physical examination, investigation of body composition and balance test may see early changes in body function and thereby prevent falls in patients with CKD.

This preliminary study clarified the relationship between edema and body function, particularly static and dynamic body balance in patients with CKD.

## Materials and methods

This study is a single-center, cross-sectional study. Patients with CKD (aged ≥40 years) who could maintain a standing position were enrolled between September 26, 2016, and September 30, 2017. We determined those with CKD as defined by the 2012 CKD guideline of the Japanese Society of Nephrology. We excluded patients who had difficulty in holding a standing position or walking and those who could not give consent. We excluded cases in which the cause of edema was heart failure, lymphedema, or hypothyroidism.

Basic characteristics and laboratory data

The basic characteristics were sex, age, blood pressure, body mass index (BMI), cause of CKD, and medications. The laboratory data were estimated glomerular filtration rate (eGFR), hemoglobin (Hb), and C-reactive protein (CRP). The laboratory data were obtained within five days of their physical function assessments.

Edema and muscle mass

The body fluid volume and muscle mass were measured using the InBody S10 (InBody Japan Inc., Tokyo, Japan). We performed these measurements between 4 p.m. and 5 p.m. in all cases. The Inbody S10 provided us with the total body water (TBW), intracellular water (ICW), extracellular water (ECW), ECW/TBW ratio, and skeletal muscle mass. The ratio of ECW/TBW, 0.38 is considered for healthy individuals [11]. The usual range of ECW/TBW is between 0.36 and 0.4. Values of 0.4 and higher were defined as “edema.”

The physical assessment of lower extremity edema was performed using the method described by Fukazawa et al. [12] This was performed before using the InBody S10.

Static balance while standing

The center of gravity sway was measured using the Kinetogravicorder 7100 (Anima Inc., Tokyo, Japan) as per a report by Hirase et al. [13]. The result of this is expressed as total trajectory length.

Dynamic balance while standing

The index of postural stability (IPS) was measured using the Kinetogravicorder 7100 as dynamic balance while standing. This was done according to a report by Mochizuki et al. [14].

Measuring dynamic balance during motion

The timed up & go test (TUG) was used to predict falls. This measurement was performed according to a report by Nakatani et al. [15]. Measurements were taken twice, and the faster time of the two was used.

Measuring knee extension muscle strength

We measured this by using the Micro Total Analysis System (μTas) F-1 (Anima Inc., Tokyo, Japan) as per a report by Katoh et al. [16]. We added TUG after starting this study. Thus, the number of knee extension muscle strength measurements was 28, and the number of TUG was 21.

Assessment of nutritional status

The geriatric nutritional risk index (GNRI) is calculated as [1.489 x albumin (g/L)] + [41.7 x (weight/ ideal weight)]. Ideal weight is calculated differently for men and women. For men：height (cm) - 100 - {[height (cm) - 150] /4} , for women：height (cm) - 100 - {[height(cm) - 150] /2.5}. In the original method, four grades are defined: high risk (GNRI: <82), moderate risk (GNRI: 82 to <92), low risk (GNRI: 92 to ≤98), no risk (GNRI: >98) [17].

Functional independence measure

We measured the functional independence measure (FIM) as an assessment of the activities of daily living (ADL). We assessed this by observing the activities of daily living during their hospitalization.

All subjects were provided both written and oral descriptions of the study, and their written consent was obtained. This study was approved by the Institutional Review Board of the Department of Medicine, Oita University (approval no. 1098). Per the Declaration of Helsinki, the anonymity of the subjects was ensured.

Statistical analysis

Parameters are presented as mean ± standard deviation (SD) or median according to the distribution as appropriate. We analyzed the differences between the non-edema group versus the edema group by using a non-paired t-test or Mann-Whitney U test and χ-squared test for categorical variables. The relation between edema and physical function was analyzed using Pearson’s correlation coefficient or Spearman’s rank correlation coefficient if a normal distribution was not observed. We conducted a multivariate regression analysis to investigate the factors related to knee extension muscle strength and dynamic balance in motion (TUG).

Due to the small number of cases, knee extension strength was divided into three models: model 1 (age, sex, ECW/TBW), model 2 (age, sex, TUG), model 3 (age, sex, albumin (Alb)), and each of the confounding factors were examined separately as previously reported [18]. The TUG was divided into four models: model 1 (age, sex, ECW/TBW), model 2 (age, sex, IPS), model 3 (age, sex, GNRI), model 4 (age, sex, Hb), and each of the confounding factors was examined separately. Statistical analysis was performed using Statistical Package for Social Sciences (SPSS) version 22 (IBM Corp., Armonk, NY, USA). A p-value <0.05 was considered statistically significant.

## Results

Basic characteristics of this study, comparison of edema and non-edema groups

A comparison of edema and non-edema groups is shown in Table 1. Participants of this study include 20 men and 10 women (mean age, 71.6 ± 9.0 years). Two patients refused the knee extension muscle strength test. The cause of CKD was diabetes mellitus in 17 cases; nephrosclerosis in five cases; immunoglobulin A (IgA) glomerulonephritis and nephrotic syndrome in two cases; membranous nephritis, membranoproliferative glomerulonephritis, antineutrophil cytoplasmic antibody (ANCA)-associated nephritis, unknown of origin in one case. According to CKD stage, stage 1 was zero, stage 2 was seven, stage 3 was six, stage 4 was six, and stage 5 was eleven.

**Table 1:** Characteristics of participants (20 men and 10 women) Values are presented as a mean ± standard deviation for continuous variables with normal distribution and a median (inter-quartile range) for continuous variables with skewed distribution. BMI: Body mass index, eGFR: Estimated glomerular filtration rate, Hb: Hemoglobin, Alb: Albumin, CRP: C-reactive protein, IPS: Index of postural stability, TUG: Timed up & go test, FIM: Functional independence measure, CKD: Chronic kidney disease, GNRI: Geriatric nutritional risk index, IgA: Immunoglobulin A, ANCA: Antineutrophil cytoplasmic antibody

Variables	Edema (ECW/TBW >0.4) N=20	Non-edema (ECW/TBW <0.4 N=10	p-value
Men (n, %)	13 (65)	7 (35)	0.560
Age (years)	74.1 ± 8.7	66.8 ± 9.3	0.029
BMI (kg/m^2^)	23.4 ± 3.9	22.9 ± 4.1	0.778
eGFR (mL/min/1.73m^2^)	16.6 (11.5-48.9)	39.5 (7.7-73.0)	0.379
Alb (g/dl)	3.0 ± 0.6	3.8 ± 0.3	0.000
Hb (g/dl)	10.6 ± 2.1	12.9 ± 1.7	0.000
CRP (mg/dl)	0.1 (0.04-0.79)	0.05 (0.03-0.45)	0.365
GNRI	84.7±10.8	97.0±5.8	0.000
Total trajectory length (cm)	51.2 (43.7-75.1)	51.1 (39.7-89.2)	0.895
IPS	1.1 ± 0.5	1.3 ± 0.4	0.166
Knee extension muscle strength (Nm/kg)	1.1±0.4	1.6 ± 0.6	0.039
TUG (seconds)	8.5 ± 1.6	6.2 ± 1.7	0.007
Muscle mass (kg)	40.0 ± 9.4	41.3 ± 8.7	0.524
Edema score (point)	4.2 ± 2.9	1.8 ± 1.7	0.008
FIM (point)	125.5 (122.3-126)	126 (126)	0.056
Steroid (n, %)	0 (0)	1 (10)	0.333
Diuretic (n, %)	12 (60)	3 (30)	0.245
Antihypertensive (n, %)	17 (85)	6 (60)	0.143
Primary disease			
Diabetic kidney disease (n, %)	13 (65)	4 (40)	0.181
Nephrosclerosis (n, %)	3 (15)	2 (20)	0.551
IgA glomerulonephritis (n, %)	0 (0)	2 (20)	0.103
Nephrotic syndrome (n, %)	2 (10)	0 (0)	0.437
Membranous nephritis (n, %)	1 (5)	0 (0)	0.667
Membranoproliferative glomerulonephritis (n, %)	1 (5)	0 (0)	0.667
ANCA (n, %)	0 (0)	1 (10)	0.333
Unknown (n, %)	0 (0)	1 (10)	0.333
CKD stage			0.437
CKD stage 2 (n, %)	3 (15)	4 (40)	
CKD stage 3 (n, %)	4 (20)	2 (20)	
CKD stage 4 (n, %)	5 (25)	1 (10)	
CKD stage 5 (n, %)	8 (40)	3 (30)	

Antihypertensive drugs were prescribed to 77% of the patients (angiotensin-converting-enzyme(ACE) inhibitors (n=2), angiotensinⅡreceptor blockers (ARB) (n=5), calcium antagonists (n=22), alpha-blockers (n=3), beta-blockers (n=1), and alpha-beta-blockers (n=1)). Fifteen patients had diuretics. The edema group used diuretics in 60% of cases, and the non-edema group used them in 30% of cases. On the other hand, the edema group used antihypertensive drugs in 85% of cases, and the non-edema group used them in 60% of cases. Steroid drugs in 10% of cases of the non-edema group were used. The edema group was older and had lower Alb and Hb values than the non-edema group. The edema group had a higher edema score, weaker knee extension muscle strength, and slower TUG. Total trajectory length (static balance during static standing) and IPS (dynamic balance while standing) were not significantly different between the two groups. Although, TUG and knee extension muscle strength were significantly different between the two groups. The usage of antihypertensive drugs and diuretics was the same between the two groups.

Relationship between edema, age, laboratory date, GNRI, and physical functions

Correlation of various factors are shown in Table 2. No correlation was found between edema (ECW/TBW) and age or body balance as total trajectory length or IPS (r=0.243, p=0.197; r= -0.122, p=0.52; r=0.087, p=0.65, respectively). A poor inverse correlation existed between edema (ECW/TBW) and knee extension muscle strength (r= -0.395, p=0.038). A moderate positive correlation existed between edema (ECW/TBW) and dynamic body balance in motion (TUG) (r=0.572, p=0.007). A moderate negative correlation existed between edema (ECW/TBW) and Alb or Hb or GNRI (r= -0.745, p<0.001; r= -0.419, p=0.021; r= -0.699, p<0.001, respectively).

**Table 2:** Correlation between edema, age, laboratory date, GNRI, and physical function ECW/TBW: Extracellular water/total body water, eGFR: Estimated glomerular filtration rate, Alb: Albuminm, Hb: Hemoglobin, GNRI: Geriatric nutritional risk index, TUG: Timed up & go test, IPS: Index of postural stability Spearman's rank correlation coefficient *p<0.05; **p<0.01

Variables	ECW/TBW	Edema scale	Age	eGFR	Alb	Hb	GNRI	Knee extension muscle strength	TUG	IPS	Total trajectory length
ECW/TBW		0.605**	0.243	-0.151	-0.745**	-0.419*	-0.699**	-0.395*	0.572**	-0.087	-0.122
Edema scale	0.605**		-0.073	-0.286	-0.515**	-0.036	-0.422*	-0.352	0.408	-0.141	0.103
Age	0.243	-0.073		-0.096	-0.26	-0.388*	-0.333	-0.308	0.503*	-0.394*	0.002
eGFR	-0.151	-0.286	-0.096		0.25	0.436*	0.119	0.319	-0.612**	0.145	-0.240
Alb	-0.745**	-0.515**	-0.26	0.25		0.471**	0.964**	0.327	-0.511	0.063	-0.018
Hb	-0.419*	-0.036	-0.388*	0.436*	0.471**		0.455*	0.308	-0.618**	0.181	-0.007
GNRI	-0.699**	-0.422*	-0.333	0.119	0.964**	0.455*		0.282	-0.455**	0.006	0.055
Knee extension muscle strength	-0.395*	-0.352	-0.308	0.319	0.327	0.308	0.282		-0.764**	0.243	-0.211
TUG	0.572**	0.408	0.503*	-0.612**	-0.511	-0.618**	-0.455**	-0.764**		-0.498*	0.404
IPS	-0.087	-0.141	-0.394*	0.145	0.063	0.181	0.006	0.243	-0.498*		-0.385*
Total trajectory length	-0.122	0.103	0.002	-0.240	-0.018	-0.007	0.055	-0.211	0.404	-0.385*	

Multivariate regression analysis

Multivariate regression analysis results are shown in Tables 3, 4. Knee extension muscle strength was associated with edema (ECW/TBW; lower extremes) and TUG after adjusted for age and sex (β= -0.337, p= 0.044, 95% CI= -19.604 to -0.297; β= -0.576, p= 0.006, 95% CI= -0.242 to -0.046). The TUG was also associated with edema (ECW/TBW; lower extremes) after adjusted for age and sex (β= 0.416, p= 0.046, 95% CI = 0.896 to 93.130). The TUG was not associated with IPS and GNRI and Hb (β= -0.433, p= 0.059, 95% CI= -5.007 to -0.101; β= -0.191, p= 0.385, 95% CI= -0.114 to 0.046; β-0.404, p= 0.077, 95% CI= -0.732 to 0.042, respectively).

**Table 3:** Multiple regression analysis of factors related to knee extension muscle strength Each multivariate analysis was conducted by using age and sex plus one factor as confounding factors to avoid overfitting due to the small sample size. ECW/TBW: Extracellular water/total body water, TUG: Timed up & go test, Alb: Albumin

Confounding factor	Coefficient of determination (R^2^)	Standardized coefficient (β)	p-value	95% confidence interval
Knee extension on muscle strength adjusted by age, sex, and ECW/TBW	0.393	-0.337	0.044	-19.604 to -0.297
Knee extension on muscle strength adjusted by age, sex, and TUG	0.548	-0.576	0.006	- 0.242 to -0.046
Knee extension on muscle strength adjusted by age, sex, and Alb	0.339	0.235	0.152	- 0.074 to 0.449

**Table 4:** Multiple regression analysis of factors related to TUG Each multivariate analysis was conducted by using age and sex plus one factor as confounding factors to avoid overfitting due to the small sample size. TUG: Timed up & go test, ECW/TBW: Extracellular water/total body water, IPS: Index of postural stability, GNRI: Geriatric nutritional risk index, Hb: Hemoglobin

Confounding factor	Coefficient of determination (R^2^)	Standardized coefficient (β)	p-value	95% confidence interval
TUG adjusted by age, sex, and ECW/TBW	0.389	0.416	0.046	0.896 to 93.130
TUG adjusted by age, sex, and IPS	0.374	-0.433	0.059	-5.007 to -0.101
TUG adjusted by age, sex, and GNRI	0.258	-0.191	0.385	-0.114 to 0.046
TUG adjusted by age, sex, and Hb	0.357	-0.404	0.077	-0.732 to 0.042

## Discussion

This study's results suggest that edema may influence knee extension muscle strength. Patients with CKD are more likely to have fluid imbalances and are at risk of developing sarcopenia and frailty [19]. In addition, lower extremity muscle weakness is a predictor of future falls [20], and it has been reported that patients are at risk for falling if they do not have knee extension muscle strength of at least 1.2 Nm/kg [21]. The results of this study suggest that edema may affect knee extensor muscle strength. Therefore, the use of edema as a risk factor for falls may be a useful indicator for predicting physical function decline and future falls.

In this study, muscle mass assessed by the InBody S10 was not significantly different between the edema and non-edema groups. Since edema occurs within skeletal muscle and in subcutaneous and interstitial regions, it is likely that excess water accumulation is also incorporated into muscle mass measurements [22]. Specifically, muscle mass measured using the InBody device is thought to include the water content of the muscle in addition to the actual muscle mass itself. This may be one of the reasons why no significant differences were found between the muscle mass measurements of the two groups.

No differences were found between the two groups in total trajectory length or IPS, which are indicators of static balance during standing. Since compensatory movements of the knees and trunk in addition to the legs are used in a complex manner to maintain posture during static standing, the effects of edema and differences in knee extensor strength on static balance are considered negligible.

This study has some limitations. First, we did not investigate daily habits of walking and exercise and previous history of falls. Second, the sample size was small. Third, the muscle mass assessment may be overestimated by using the InBody device.

## Conclusions

In this study, we examined the association between edema and physical function. Patients with CKD who have edema may have decreased knee extensor strength and body balance function. The presence or absence of lower extremity edema on physical examination may be useful in predicting a functional decline in CKD patients.
